# 

**Published:** 1967-01

**Authors:** 


					January 1967
-NATIONAL LIBRARY OF MEDICINE
RECD- JAN 1 8 1967
SHELVED IN RR: YES NO.X...
CALL NO.: v .
^QPY NO. \
VI 3 "br
VCL-T--: ^ 3
DATE: /\/J b
Ss. - - X: :
'NQN-D5PTL ; ? X*
2CZ
INCORPORATING the medical journal of the south west
Vol 82 (i) No 303
J ? i 186?

				

